# Nrf2-Independent Anti-Inflammatory Effects of Dimethyl Fumarate: Challenges and Prospects in Developing Electrophilic Nrf2 Activators for Neurodegenerative Diseases

**DOI:** 10.3390/antiox13121527

**Published:** 2024-12-13

**Authors:** Yasuhiko Izumi, Yutaka Koyama

**Affiliations:** Laboratory of Pharmacology, Kobe Pharmaceutical University, 4-19-1 Motoyamakita-machi, Higashinada-ku, Kobe 658-8558, Japan; koyama-y@kobepharma-u.ac.jp

**Keywords:** electrophilic Nrf2 activators, antioxidant effect, anti-inflammatory effect, dimethyl fumarate, monomethyl fumarate

## Abstract

The NF-E2-related factor 2 (Nrf2)-antioxidant response element (ARE) pathway is a potential therapeutic target for central nervous system diseases. This review emphasizes the role of oxidative stress and neuroinflammation in neurodegenerative diseases, highlighting the therapeutic potential of Nrf2 activators such as dimethyl fumarate (DMF). DMF, initially administered for treating psoriasis, has demonstrated efficacy in multiple sclerosis and is metabolized to monomethyl fumarate, which may exert significant therapeutic effects. DMF activates the Nrf2-ARE pathway, and recent studies have indicated that its anti-inflammatory effects occur through Nrf2-independent mechanisms. Electrophilic Nrf2 activators, such as DMF, covalently bind to cysteine residues in proteins and modulate their function. We discuss the implications of cysteine residue modifications by DMF, which may cause both therapeutic benefits and potential off-target effects. Furthermore, we propose a chemical proteomics-based drug discovery approach to achieve desired therapeutic effects by selectively covalently modifying cysteines in target proteins. These findings advocate for a broader understanding of the Nrf2-independent mechanisms of electrophilic Nrf2 activators, thereby improving drug discovery strategies that target neurodegenerative diseases while minimizing toxicity.

## 1. Introduction

Oxidative stress has increasingly been found to be involved in the pathogenesis of different neurodegenerative diseases, including Alzheimer’s disease (AD) [1], Parkinson’s disease (PD) [2], multiple sclerosis (MS) [3], amyotrophic lateral sclerosis (ALS) [4,5], Huntington’s disease (HD) [6], and Friedrich’s ataxia [7]. Antioxidant-related therapies are currently being explored as promising potential treatments, considering the pivotal role of oxidative stress in these conditions.

These neurodegenerative diseases are frequently characterized by an accompanying inflammatory response, which exacerbates cellular damage. Neuroinflammation, when coupled with oxidative stress, represents a crucial aspect that needs to be considered in neurodegenerative disease progression [8]. The interplay between oxidative stress and neuroinflammation may develop a vicious cycle, contributing to neuronal dysfunction and death. Therefore, targeting both oxidative stress and inflammation is a valuable strategy for establishing effective therapeutic interventions for these neurodegenerative disorders.

**Figure 1:**
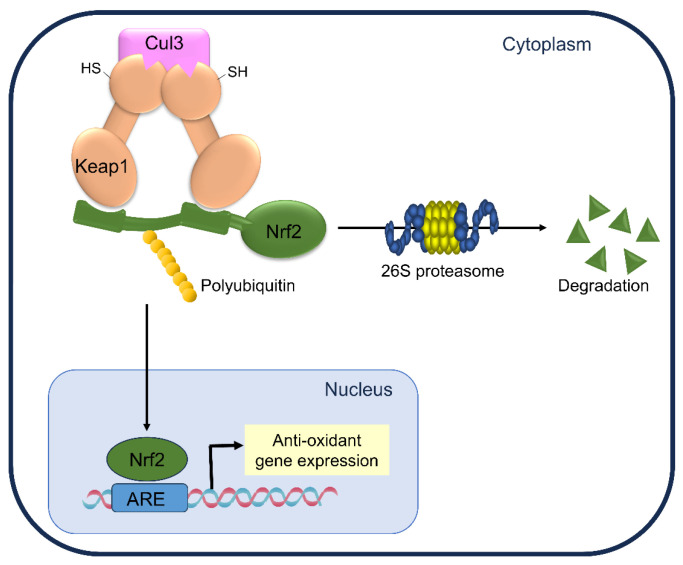
Schematic representation of the Keap1-Nrf2-ARE pathway.

Cells must maintain cellular redox homeostasis to overcome oxidative stress. The endogenous antioxidant capacity of cells is regulated by activating the transcription factor NF-E2-related factor 2 (Nrf2). Nrf2 forms a complex with Kelch-like ECH-associated protein 1 (Keap1) in the absence of oxidative stress, causing its ubiquitination and degradation via Cullin 3-based E3 ubiquitin ligase (Cul3), which suppresses its function. Modification of specific Keap1 cysteine thiols induces a conformational distortion of Keap1 and inhibits Nrf2 ubiquitination upon exposure to oxidative stress or electrophilic agents. Consequently, the Nrf2 protein accumulates and its translocation into the nucleus is promoted. Nrf2 binds to the antioxidant response element (ARE) once in the nucleus, thereby regulating phase II detoxification enzymes and antioxidant protein expressions [9] (Figure 1).

The genes activated through the Nrf2-ARE pathway are diverse, encompassing components of the redox regulation system (e.g., superoxide dismutase, catalase, and thioredoxin), glutathione synthesis and metabolism (e.g., glutathione peroxidase, glutathione reductase, glutamate-cysteine ligase, and glutathione synthase), quinone recycling (e.g., NAD[P]H quinone oxidoreductase), and iron homeostasis (e.g., heme oxygenase-1 (HO-1) and ferritin). Other regulated pathways involve the pentose phosphate pathway (e.g., glucose-6-phosphate dehydrogenase and phosphogluconate dehydrogenase), nucleotide biosynthesis (e.g., phosphoribosyl pyrophosphate amidotransferase and methylenetetrahydrofolate dehydrogenase 2), serine/glycine biosynthesis (e.g., 3-phosphoglycerate dehydrogenase and phosphoserine aminotransferase 1), amino acid metabolism (e.g., glutaminase and cystine-glutamate antiporter), NADPH production (e.g., malic enzyme 1 and isocitrate dehydrogenase 1), and lipid biosynthesis (e.g., fatty acid synthase) [10,11].

In neurodegenerative diseases, Nrf2 expression is altered, and genetic manipulation of Nrf2 has been reported to either improve or exacerbate pathological models (Table 1). Therefore, Nrf2-activating compounds may serve as promising therapeutic agents for neurodegenerative diseases related to oxidative stress and inflammation [12,13,14]. Indeed, Nrf2 activators, such as dimethyl fumarate (DMF) and omaveloxolone, have been administered to treat MS and Friedrich’s ataxia, respectively. The mechanisms of action of Nrf2 activators remain poorly understood despite their success in clinical therapy. Majkutewicz (2022) [15], in an earlier review, discussed the mechanisms by which DMF exerts cytoprotective and anti-inflammatory effects in preclinical models of neurodegenerative diseases. However, recent advancements in research have increasingly determined Nrf2-independent target proteins involved in the anti-inflammatory action of DMF. Herein, we summarize the Nrf2-independent mechanisms of the anti-inflammatory effects of Nrf2 activators and discuss their potential future applications in neurodegenerative disease treatment.

## 2. History of Human Use of Fumaric Acid Ester

DMF was first used in autoimmune disorder treatment more than fifty years ago. Psoriasis, which is an inflammatory skin condition characterized by epidermal hyperplasia and inflammatory cell infiltration into the skin lesions, became the focus of this pioneering treatment. In 1959, Schweckendiek, a physician, self-administered fumaric acid, a compound that serves as an intermediate in the citric acid cycle, in an attempt to alleviate his psoriasis symptoms [33]. By around 1990, a mixture that contained DMF and monoethyl fumarate (MEF) demonstrated beneficial effects in treating psoriasis, as highlighted in the study by Nieboer et al. (1989) [34]. Based on these encouraging results, the fumaric acid ester mixture was officially approved for treating psoriasis in Germany in 1994. Among the components of this mixture, DMF is considered the principal active ingredient responsible for its therapeutic effects.

Further research subsequently revealed that DMF was effective in treating MS, with studies confirming its efficacy [35,36]. In 2013, the United States Food and Drug Administration (FDA) approved a medication that contains only DMF for treating relapsing-remitting MS. Additionally, in 2017, DMF received approval from the European Medicines Agency for treating psoriasis.

Crucially, DMF exhibits immune-modulatory properties without causing significant immune suppression, making it a safe and effective long-term treatment option for patients with psoriasis [37]. This dual capability—modulating the immune response while minimizing adverse effects—has contributed to DMF’s acceptance and use in clinical practice for these disorders. Conversely, DMF was considered one of the drugs associated with cognitive adverse events based on disproportionality analysis of the FDA Adverse Event Reporting System database [38]. Additionally, a few cases of progressive multifocal leukoencephalopathy related to lymphopenia have been reported in patients with MS being treated with DMF [39].

## 3. Pharmacokinetics of DMF

DMF is recognized for its effectiveness in treating both peripheral immune diseases and central nervous system-related immune disorders, indicating that its immunomodulatory and anti-inflammatory properties play crucial roles in its therapeutic effects. However, the detailed mechanisms underlying these actions remain unclear. One of the challenges in elucidating these mechanisms originates from the highly reactive nature of DMF, which is rapidly and extensively metabolized in vivo to monomethylfumarate (MMF) or rendered inactive through glutathione (GSH) conjugation [40,41].

**Figure 2:**
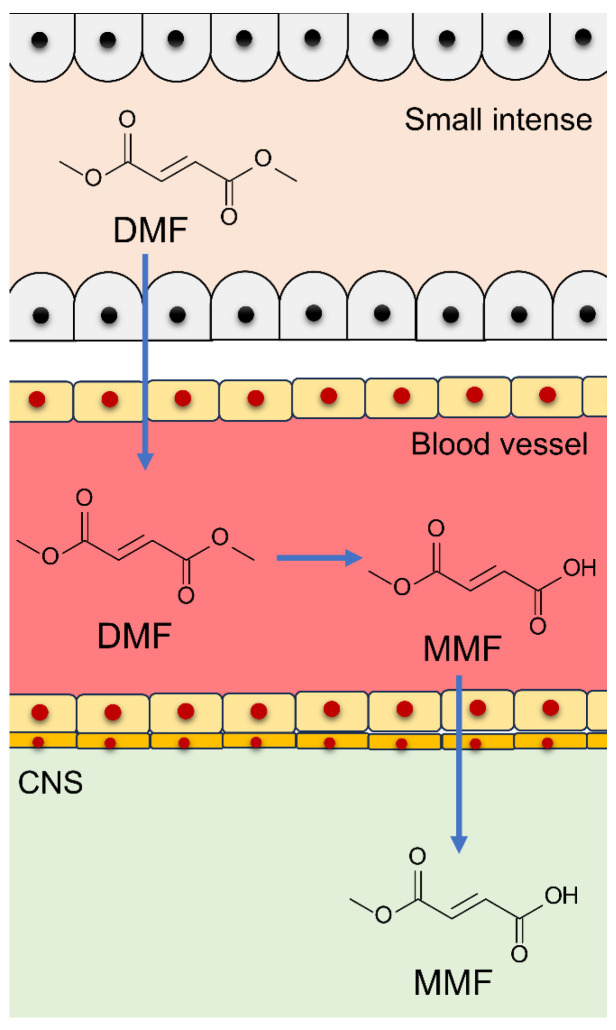
DMF pharmacokinetics. Orally administered DMF is absorbed in the small intestine, but it is rapidly metabolized by esterase to MMF, which circulates through the blood and reaches the central nervous system.

The blood concentrations of DMF itself are typically below the detection limit when DMF is administered orally to humans, whereas those of its metabolite MMF significantly increase [42,43]. This indicates that MMF is the primary active compound present in blood circulation after DMF administration. Additionally, studies have revealed that MMF penetrates the brain tissue of mice that have received oral DMF, indicating its potential central effects [44] (Figure 2).

MMF emerges as the primary active molecule in vivo after DMF administration from a pharmacokinetic perspective. This distinction is crucial for understanding the therapeutic effects attributed to DMF. Various mechanisms of action have been proposed to explain DMF’s therapeutic effects, whereas exercising caution, particularly in studies utilizing cell cultures, is important. This is because the effects observed in vitro may not accurately reflect the in vivo actions of DMF, considering that oral administration causes rapid DMF metabolism into MMF.

## 4. Effect of DMF on Experimental Autoimmune Encephalomyelitis (EAE)

EAE serves as an acute autoimmune model for MS. A deficiency in the transcription factor Nrf2 exacerbates EAE development, indicating that Nrf2 activation mitigates the pathogenesis of MS [24]. Either oral administration of DMF or its metabolite MMF has demonstrated therapeutic effects in the EAE model, which are associated with the anti-inflammatory actions of macrophages [45]. Notably, MMF has demonstrated superior efficacy compared to DMF when administered at a similar dose. DMF has been shown to promote the generation of type II dendritic cells, which subsequently induce T helper 2 (Th2) cells, in the EAE model, in addition to its effects on macrophages [46]. Linker et al. (2011) [47] demonstrated that DMF exhibited protective effects in the EAE model and that this effect was suppressed in Nrf2 knockout mice. However, Nrf2 deficiency alters the baseline symptoms in EAE model mice; thus, ascertaining whether the protective effects of DMF are Nrf2-dependent remains challenging. Severity-matched experiments in a more precisely controlled EAE model later demonstrated that DMF retained its protective effects, regardless of Nrf2 deficiency [44]. These discrepancies may be because the former assessed the chronic phase of the EAE model, whereas the latter evaluated the acute phase. In any case, the protective effects of DMF in the EAE model are indicated to have an Nrf2-independent component.

Interestingly, MMF, but not DMF, has been determined as a potent agonist of hydroxycarboxylic acid receptor 2 (HCA2; GPR109A) [48,49]. Activation of this receptor decreases lipolysis in adipocytes and increases prostaglandin formation in keratinocytes [50]. Among immune system cells, HCA2 is predominantly expressed in macrophages, monocytes, and neutrophils, but is not significantly present in T cells [48]. HCA2 stimulation by MMF inhibits microglial activation through nuclear factor-κB (NF-κB) pathway inhibition, mediated via the AMP-activated protein kinase (AMPK)/NAD+-dependent protein deacetylase sirtuin-1 (SIRT1) axis [51]. Oral DMF administration in the EAE model has been shown to reduce neutrophil infiltration into the central nervous system, as well as neurological damage and demyelination in wild-type mice. However, these protective effects were not demonstrated in HCA2-deficient mice [52]. These results indicate that the effects of DMF in the EAE model are primarily mediated through HCA2 stimulation by MMF [53].

## 5. Differences in the In Vitro Effect of Fumaric Acid Ester

Several studies have revealed that DMF demonstrates greater efficacy than MMF in in vitro settings. Pre-incubation with DMF was shown to protect hippocampal HT22 cells from oxidative glutamate toxicity from a cytoprotective standpoint, whereas MMF did not provide the same protection level [54]. Similarly, in another study, DMF pre-treatment preserved cellular viability against amyloid-beta-induced toxicity much more effectively than MMF [55]. Regarding anti-inflammatory effects, DMF exhibited significant inhibition of pro-inflammatory cytokine production by peripheral blood mononuclear cells, an effect that MMF did not replicate [56]. Furthermore, DMF inhibited T-cell activation [57] and reduced cytokine release from activated dendritic cells, whereas MMF did not demonstrate these effects [58]. Notably, short-term DMF exposure was sufficient to suppress macrophage activation, a response not observed with MMF [59]. Both DMF and MMF suppressed similar patterns of biomarkers in multiple in vitro human disease models. However, DMF was significantly more potent overall [56].

**Figure 3:**
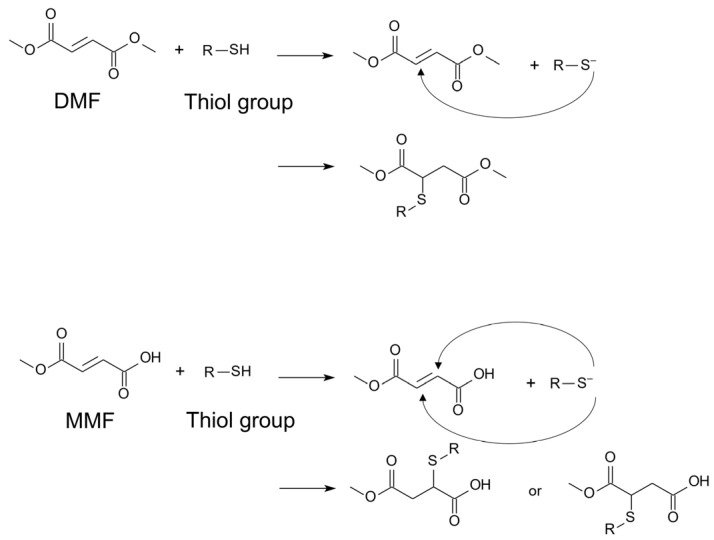
Chemical reactions of DMF and MMF with thiol groups. DMF and MMF contain α,β-unsaturated carbonyl groups, enabling them to act as Michael acceptors and form covalent adducts with thiol groups (e.g., cysteine residues).

The chemical structures of DMF and MMF are one factor contributing to their differences in efficacy. Both compounds possess α,β-unsaturated carbonyl groups, enabling them to react spontaneously with thiols through a Michael-type addition reaction (Figure 3). However, MMF reacts at a considerably lower rate than DMF [59,60]. Additionally, DMF readily diffuses across the plasma membrane due to its neutral charge, whereas negatively charged MMF is unable to do so [61]. Consequently, DMF binds to multiple intracellular cysteine residues, whereas MMF demonstrates limited capacity for such binding [57].

Keap1 is one of the most well-characterized proteins that DMF binds to. Among the various cysteine residues in Keap1, C151 is considered the most reactive toward electrophiles [62,63]. Mass spectrometry studies have verified that DMF, MEF, and MMF all preferentially form covalent bonds with C151 of Keap1, determining it as the most crucial site for alkylation by fumaric acid esters [47,63,64]. The differences in their chemical characteristics cause distinct outcomes, despite these compounds both targeting C151. Experiments using cultured cells have indicated a more pronounced reactivity of DMF to Keap1 than that of MEF [63]. DMF strongly activates the Nrf2-ARE pathway, whereas the efficacy of MMF or MEF in this regard is markedly inferior [63,65].

## 6. The Relationship Between Inflammation, Oxidative Stress, and Nrf2

Reactive oxygen species (ROS) have been determined as crucial mediators of inflammatory signals, particularly through their regulation of the Toll-like receptor 4 (TLR4)-nuclear factor kappa-light-chain-enhancer of activated B cells (NF-κB) pathway. Bacterial lipopolysaccharide (LPS) and interferon-gamma (IFN-γ) induce NADPH oxidase expression, thereby generating superoxide anions [66]. ROS promotes TLR4 translocation to the cell membrane [67] and activates NF-κB by stimulating IκB kinase (IKK) through spleen tyrosine kinase (Syk) [68]. Additionally, ROS influence inflammatory cytokine expression via the hypoxia-inducible factor (HIF)-1α pathway, which becomes activated in response to increased ROS levels. Increased mitochondrial ROS levels have enhanced interleukin-1β (IL-1β) production through HIF-1α stabilization in LPS-activated macrophages [69]. HIF-1α directly binds to IL-1β gene’s promoter region, thereby inducing its expression [70].

Growing evidence indicates the role of Nrf2 in modulating anti-inflammatory responses, considering that the Nrf2-ARE pathway activation acts as an antioxidant [71]. Knockout of Nrf2 increases intracellular ROS levels, which improves the inflammatory response. Conversely, Nrf2 activator pre-treatment has attenuated both the increase in ROS levels and the pro-inflammatory cytokine expression in neutrophils in an Nrf2-dependent manner [72].

**Figure 4:**
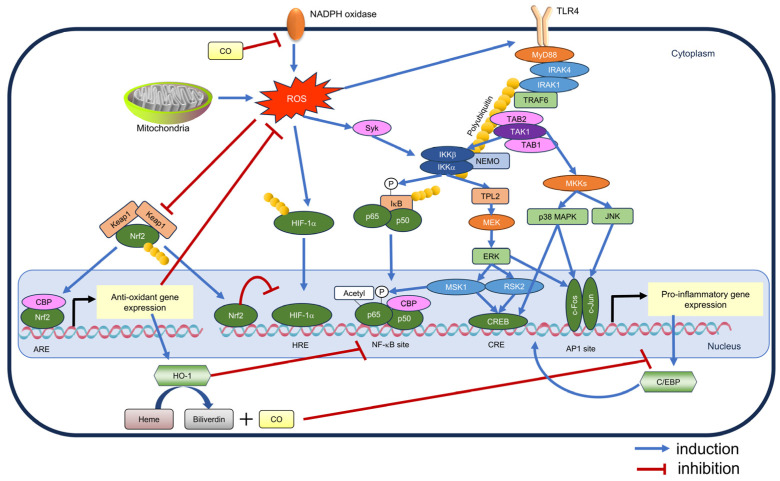
Crosstalk between the Nrf2-ARE pathway and inflammatory responses. TLR4 stimulation activates pro-inflammatory genes via the NF-κB pathway and mitogen-activated protein kinase pathway. ROS are produced by NADPH oxidase and mitochondria during the inflammatory response. ROS promote the membrane translocation of TLR4, activate IKK via syk, and induce HIF-1α accumulation, thereby promoting the inflammatory response. Conversely, ROS activate the Nrf2-ARE pathway, thereby inducing antioxidant gene expression and reducing intracellular ROS levels. HO-1, which is one of the gene products, binds to and inhibits NF-κB sites, and CO, which is a heme degradation product, inhibits pro-inflammatory transcription factor C/EBP expression and hinders NADPH oxidase. Besides, the coactivator CBP competes with Nrf2 for NF-κB, and thus the two pathways inhibit each other. Furthermore, Nrf2 binds to the promoter region of inflammatory genes and suppresses their transcriptional expression. The following abbreviations are not mentioned in the text: AP-1: activator protein-1; CRE: cAMP response element; HRE: hypoxia response element; JNK: c-Jun N-terminal kinase; NEMO: NF-κB essential modulator; TAB: TAK1-binding protein; TPL2: tumor progression locus 2.

HO-1, produced through the Nrf2-ARE pathway activation, plays a regulatory role in inflammatory responses [73]. The carbon monoxide (CO) generated from heme degradation by HO-1 hinders NADPH oxidase activity [74] and suppresses transcription factor CCAAT/enhancer binding protein (C/EBP) expression, which is crucial in inflammatory signaling [75]. Additionally, nuclear HO-1 directly reduces the DNA binding activity of NF-κB [76]. Notably, DMF-induced HO-1 inhibits IL-23 expression induction in dendritic cells [46]. Another proposed mechanism is the interplay between NF-κB and Nrf2, where these pathways may antagonize each other by competing for the transcriptional coactivator cyclic adenosine monophosphate response element binding protein (CREB)-binding protein (CBP) [77]. Interestingly, ARE sequences are found upstream of several inflammatory cytokine genes. Nrf2 generally promotes inflammatory cytokine production in non-immune cells [78], whereas Nrf2 binds to the upstream regions of inflammatory cytokine genes in an ARE-independent manner in immune cells, which ultimately inhibits the RNA polymerase II recruitment [79]. Figure 4 illustrates the crosstalk between the Nrf2-ARE pathway and inflammatory responses.

## 7. Nrf2-Independent Anti-Inflammatory Effects of Nrf2 Activators

Crosstalk between the Nrf2-ARE pathway and inflammatory responses is established, but, notably, the anti-inflammatory effects of Nrf2 activators are not exclusively mediated through this pathway. A certain study demonstrated that the anti-inflammatory effects of sulforaphane, which is an Nrf2 activator, were diminished in Nrf2 knockout macrophages when applied as a pretreatment for 6 h. However, sulforaphane demonstrated anti-inflammatory effects regardless of the presence or absence of Nrf2 when administered simultaneously [80]. In addition, simultaneous treatment with an Nrf2 activator is sufficient to achieve anti-inflammatory effects against IFN-γ independent of Nrf2 [81]. These findings suggest that the immediate anti-inflammatory effects of Nrf2 activators may involve an Nrf2-independent mechanism.

Indeed, investigations using Nrf2 knockout models and/or Nrf2 inhibitors have revealed that inhibiting inflammatory cytokine production by DMF operates regardless of Nrf2 in various immune cell types, including macrophages, dendritic cells, T cells, splenocytes, and microglia [56,57,58,81,82,83]. These results provide compelling evidence that the anti-inflammatory effects of DMF involve mechanisms that do not depend on Nrf2 activation. This emphasizes the complexity of the pharmacological effects of Nrf2 activators and indicates their use in therapeutic strategies that do not solely depend on Nrf2 activation.

## 8. Exploring the Nrf2-Independent Anti-Inflammatory Mechanisms of DMF

Identifying proteins other than Keap1 to which DMF directly binds is important to clarify the Nrf2-independent anti-inflammatory mechanism of DMF. Analyzing intracellular signaling pathways is crucial for narrowing down the target proteins. Several studies have examined the intracellular signaling pathways affected by DMF. Notably, DMF is not associated with inhibitory kappa B (IκB) degradation; rather, it inhibits the Ser276 phosphorylation of NF-κB by targeting mitogen- and stress-activated kinase 1 (MSK1). Ultimately, this inhibition suppresses NF-κB’s DNA binding activity in airway smooth muscle cells [84]. MSK1 is considered a key nuclear kinase responsible for the Ser276 phosphorylation of the NF-κB p65 subunit [85]. Furthermore, DMF hinders cell proliferation in keratinocytes by blocking both MSK1 and 90 kDa ribosomal S6 kinase (RSK1) activation in the short term [86]. Both MSK1 and RSK1 are downstream kinases of extracellular signal-regulated kinases 1 and 2 (ERK1/2).

Additionally, Peng et al. (2012) [87] revealed that DMF reduces cytokine production from dendritic cells by inhibiting ERK, NF-κB, and MSK1 phosphorylation. These results indicate that DMF exhibits anti-inflammatory effects not only by inhibiting NF-κB but also by affecting a broader range of NF-κB-related signaling proteins. Supporting this notion, Kastrati et al. (2016) [88] indicated that DMF directly binds to NF-κB p65, thereby inhibiting its activity through an alkynyl-modified probe designed for visualization and immunoprecipitation via Click chemistry. Moreover, Andersen et al. (2018) [89] revealed that DMF directly interacts with cysteine residues in the RSK2 protein using radioactive labeling techniques.

In contrast, McGuire et al. (2016) [82] demonstrated that DMF blocked IKKβ and ERK1/2 phosphorylation and IκB and interleukin receptor-associated kinase 1 (IRAK1) degradation in response to LPS in macrophages. The IRAK family plays a crucial role in propagating signals from myeloid differentiation factor 88 (MyD88) to tumor necrosis factor receptor-associated factor 6 (TRAF6) and subsequently to transforming growth factor-β-activated kinase 1 (TAK1) within TLR and IL-1 signaling pathways. These results indicate that DMF exerts an inhibitory effect at an early stage of the TLR4 signaling pathway in macrophages. Polyubiquitin chain formation is required for the interaction of IRAK with TRAF6, for IKK complex recruitment, and for TAK1 activation, which triggers mitogen-activated protein kinase cascades [90]. Proteasome-dependent protein degradation involves ubiquitin chains linked at Lys48 (K48 chains), whereas ubiquitin chains linked at Lys63 (K63 chains) regulate signal transduction, including NF-κB signaling. McGuire et al. (2016) [82] showed that DMF inhibits E2 ubiquitin-conjugating enzymes through covalent modification, thereby blocking the formation of polyubiquitin chains downstream of TLR signaling.

## 9. Comprehensive Analysis of Protein Modifications by DMF

There are many other proteins that it is assumed DMF binds to. Additionally, comprehensive analysis and identification of these proteins, regardless of their involvement in the anti-inflammatory effect, is necessary to determine the effects of DMF on cell function. Fumarate, which is an intermediate of the Krebs cycle, covalently modifies cysteine residues in a process known as succination. Succinated proteins are detected using anti-S-(2-succino)cysteine antibodies and determined by mass spectrometry [91]. The cysteine residue of glycolytic enzyme glyceraldehyde 3-phosphate dehydrogenase (GAPDH) is one of the primary targets of this modification [92]. Kornberg et al. (2018) [93] revealed that both DMF and monomethyl fumarate (MMF) succinate and inactivate the catalytic cysteine of GAPDH, thereby downregulating aerobic glycolysis in activated myeloid and lymphoid cells, which mediates their anti-inflammatory effects. Additionally, Piroli et al. (2014) [94] indicated that the polymerization of tubulin, which is the primary succinated protein, is hindered through modification by DMF. Furthermore, Piroli et al. (2019) [95] demonstrated that ester group removal by saponification increases DMF-modified protein detectability using anti-S-(2-succino)cysteine antibodies and determined 24 proteins modified by DMF in neurons and astrocytes, including cofilin 1, tubulin, and collapsin response mediator protein 2 (CRMP2).

**Figure 5:**
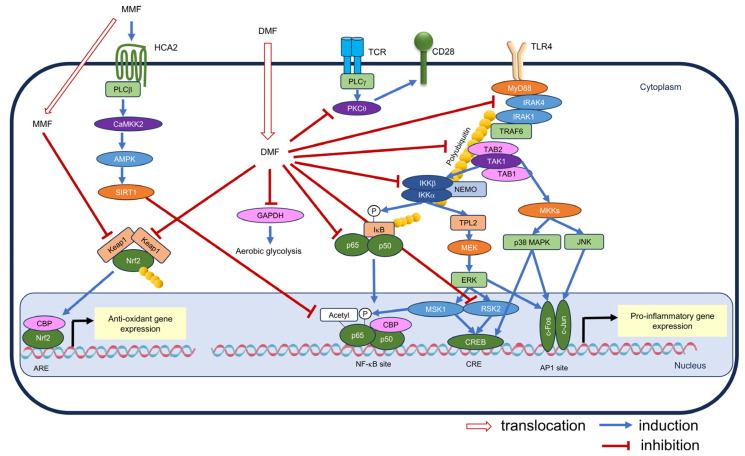
Mechanisms of anti-inflammatory effects of DMF and MMF in cells. DMF penetrates the cell membrane and activates the Nrf2-ARE pathway by modifying Keap1. DMF binds to IRAK4 and hinders its interaction with MyD88, and it binds to PKCθ in T cells and inhibits its interaction with CD28. Additionally, DMF binds to and inhibits E2 ubiquitin-conjugating enzymes, thereby suppressing the formation of linear ubiquitin chains required for inflammatory responses. DMF binds to the p65 subunit of NF-κB, IKKβ, and RSK2 and inhibits their activity. Further, it binds to GAPDH, thereby suppressing aerobic glycolysis and inclining immune cells to an anti-inflammatory state. Conversely, MMF demonstrates a weak activation effect on the Nrf2-ARE pathway and acts as a ligand for HCA2, which promotes p65 subunit deacetylation and inhibits its activity.

Modified cysteine residues are directly determined by mass spectrometry, but the quantitative reactivity of functional cysteines remains challenging to evaluate. A chemical proteomic method, also known as isotopic Tandem Orthogonal Proteolysis-Activity-Based Protein Profiling (isoTOP-ABPP), is particularly useful to quantitatively profile the reactivity of cysteine residues to small-molecule ligands [96,97]. Using the competitive isoTOP-ABPP method, Blewett et al. (2016) [57] identified the DMF sensitivity of >2400 cysteine residues in human T cells. These include IKKβ, protein kinase C-θ (PKCθ), and tumor necrosis factor α-induced protein 3 (TNFAIP3), which are NF-κB signaling pathway components or regulators. Modification of PKCθ by DMF prevented its interaction with CD28, which is crucial for T-cell activation, but the result that DMF reduced IL-2 secretion even in PKCθ knockout T cells indicates that DMF acts through other targets to achieve its full inhibitory effect. Furthermore, Zaro et al. (2020) [58] revealed that ~170 cysteine residues demonstrated substantial sensitivity to DMF by isoTOP-ABPP in dendritic cells. In particular, the cysteine residue of IRAK4 was highly sensitive to DMF, whereas interestingly, the catalytic cysteine of GAPDH was insensitive. DMF adduction of IRAK4 does not influence its kinase activity but disrupts IRAK4 and MyD88 interaction. Hence, DMF fails to decrease TNF-α production in cells expressing IRAK4 with cysteine replaced by alanine. Figure 5 summarizes the mechanisms of the anti-inflammatory effects of DMF and MMF in cells. The specific target molecules and pathways affected by DMF may vary depending on the dose and/or cell type. 

The isoTOP-ABPP technique could be utilized to determine the target proteins of other Nrf2 activators. However, the isoTOP-ABPP method is based on the ability of a competing small-molecule electrophile to block the reactivity of cysteine residues with the electrophilic probe iodoacetamide-alkyne; thus, several limitations were observed in this method. That is, ligand modification must be highly stoichiometric and stable during sample preparation, and the modification site needs to be accessible to electrophilic probes. An example of an unstable bond is sulforaphane, which reacts with protein thiols to form thionoacyl adducts. However, thionoacyl adducts are labile to hydrolysis and transacylation reactions, complicating the identification of the site of sulforaphane adduct on Keap1 [98,99]. Additionally, bardoxolone, which is another example of a potent Nrf2 activator, reacts with nucleophiles, but the resulting adduct is unstable and decomposes back to bardoxolone [100].

## 10. Target Specificity of Nrf2 Activators

Cysteine possesses various important biochemical functions, including catalysis, redox regulation, and protein–protein interaction. Therefore, binding of electrophilic Nrf2 activators to cysteine residues other than those of Keap1 may cause off-target and toxic effects. Electrophilic Nrf2 activators frequently demonstrate a narrow therapeutic window because they modify nonspecifically cellular proteins, causing undesirable effects. The methyl ester of bardoxolone (CDDO-Me) failed a phase 3 clinical trial for treating kidney disease in patients with diabetes due to an increased risk of heart failure [101]. However, as previously mentioned, the lack of specificity may, paradoxically, be advantageous, since the anti-inflammatory effects of DMF are in part Nrf2-independent.

Strategies should be developed to enable electrophilic Nrf2 activators to selectively target proteins with high specificity to reduce toxicity. Cysteine residues not only regulate protein function but also act as sites for developing covalent drugs. Reversible binding ligands are generally desired for safety reasons in drug discovery, but recently, the use of covalent ligands has been reconsidered [102]. So-called targeted covalent inhibitors consist of affinity and electrophilic fragments [103]. These are designed to produce highly selective and long-lasting effects by reversibly interacting with the binding site of the target protein utilizing the affinity fragment and by irreversibly binding to a nucleophilic amino acid, including cysteine, of the target protein using the electrophilic fragment with a fragment-based drug discovery (FBDD) approach [91]. Covalent drugs are discovered not only through ligand-first approaches, where covalent binding is incorporated into a known reversible ligand, but also through electrophile-first approaches, which aim to find a covalent ligand from the outset.

An example of a compound discovered through electrophile-first approaches is the KRAS(G12C) inhibitor sotorasib [104]. The KRAS oncoprotein is a key mediator of intracellular signaling pathways involved in tumor cell proliferation and survival, and its G12C mutation is a frequent driver mutation. An extensive search for covalent inhibitors for this mutant cysteine was conducted, but the lack of deep surface hydrophobic pockets in the KRAS protein has complicated efforts to determine high-affinity inhibitors. Hence, a disulfide fragment-based screening method, named tethering, was used to address this challenge [105]. When a cysteine-containing protein is equilibrated with a library of disulfide-containing small molecules in the presence of a reducing agent, only compounds that have a specific affinity with the protein can be detected as the modified cysteine by mass spectrometry, even if there is little or no binding in the absence of disulfide bonds. Ostrem et al. (2013) [106] focused on acrylamide-based electrophiles that form irreversible cysteine bonds, instead of disulfide-based compounds, and revealed ligands that selectively covalently bind to the mutant cysteine residue in KRAS(G12C). This strategy is highly useful for generating site-targeted covalent ligands and may also be utilized to enhance the target selectivity of electrophilic Nrf2 activators.

## 11. Keap1–Nrf2 Protein–Protein Interaction Inhibitors

**Figure 6:**
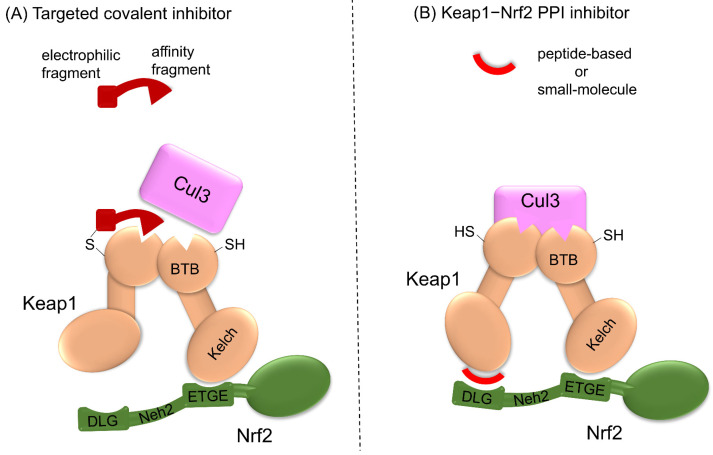
Strategies for selective Nrf2-ARE pathway activation. Modification of Keap1 cysteine residues with electrophiles is a powerful tool for activating the Nrf2-ARE pathway, but lack of selectivity causes off-target effects. (**A**) Targeted covalent inhibitors that combine electrophilic and affinity fragments may be promising compounds in the future. (**B**) Further, non-covalent PPI inhibitors that act on the interaction site of Keap1 and Nrf2 exhibit a certain degree of selectivity.

An alternative strategy for Nrf2 activators to increase specificity is to inhibit the protein–protein interaction (PPI) between Keap1 and Nrf2 non-covalently. The Neh2 domain of Nrf2 and the Kelch domain of Keap1 are involved in Nrf2–Keap1 PPI. The Neh2 domain interacts with Keap1 through two motifs: a high-affinity ETGE motif and a low-affinity DLG motif (Figure 6). To date, several peptide-based Keap1–Nrf2 PPI inhibitors that bind to those motifs have been designed, and served as useful research tool compounds [107]. Although the use of peptide-derived PPI inhibitors is limited by poor bioavailability and cell permeability, fusion proteins with a cell-penetrating peptide (cell penetrating-peptide 9 or the HIV-transactivating transcriptional activator peptide) have shown efficacy in vitro and in vivo [108,109,110].

Small-molecule Keap1–Nrf2 PPI inhibitors have been designed based on the crystal structure of the Kelch domain and developed using X-ray crystallography, structure–activity relationship studies, and fragment-based drug design (FBDD) [111]. However, a recent re-evaluation has raised questions about the properties of some reported small-molecule PPI inhibitors, highlighting the need for validation through multiple assays, including those conducted in cellular contexts [112]. Additionally, many of these compounds consisted of carboxylic acids and demonstrated large molecular sizes, which limited their ability to penetrate the central nervous system. Hence, clinically approved small-molecule Keap1–Nrf2 PPI inhibitors are unavailable, and developing more effective PPI inhibitors for clinical use remains a challenge.

Keap1–Nrf2 PPI inhibitors do not necessarily act exclusively on the Nrf2-ARE pathway. This is because several proteins bind to the Kelch domain of Keap1 [113]. The p62/SQSTM1 protein participates in autophagy by binding to both the autophagosome-localized protein LC3 and ubiquitinated proteins. Keap1–Nrf2 PPI inhibitors have induced mitophagy, which is the autophagic degradation of dysfunctional mitochondria, because p62 interacts with the Kelch domain on Keap1 [114]. Additionally, IKKβ contains both ETGE and DLG motifs, which is crucial for Keap1 interaction [115]. Keap1–Nrf2 PPI inhibitors are indicated to suppress Keap1-mediated degradation, thereby causing IKKβ accumulation and activating the NF-κB pathway [116]. Conversely, p21, which is a cyclin-dependent kinase inhibitor, binds to the DLG motif of Nrf2 and competes with Keap1 [117]. p21 is involved in many functions, including cell cycle regulation, DNA repair, and apoptosis, which may be influenced by PPI inhibitors. Moreover, p62, which is another Keap1-binding protein, competes with Nrf2 for binding to Keap1 and activates the Nrf2-ARE pathway by displacing Nrf2 from its complex with Keap1 through a non-canonical mechanism [118].

## 12. Conclusions

This review focused on the Nrf2-independent anti-inflammatory actions of DMF. Considering that orally administered DMF is converted to MMF in the bloodstream and that MMF exhibits poor cell penetration and limited capacity to modify intracellular cysteines, as well as diminished effects in the EAE model of HCA2-deficient mice, the therapeutic effect of DMF appears to primarily arise from HCA2 stimulation by its metabolite, MMF. However, the implications of DMF’s effects in cultured cells remain crucial, even with no Nrf2 involvement. This is because electrophilic Nrf2 activators may modify cysteine residues of intracellular proteins in a manner such as DMF. Isolating the aspects related to toxicity rather than focusing solely on the Nrf2-dependent effects to fully harness all their potential benefits is essential because the immunomodulatory effects of these electrophilic Nrf2 activators encompass both Nrf2-dependent and independent effects. Recent improvements in chemoproteomic technologies help meet the desired therapeutic effect while minimizing covalent off-target effects. This strategy may expand the possibilities for drug discovery using the thiol reactivity of electrophilic Nrf2 activators. Nrf2 activators are anticipated to have broader applications, as they are considered potential therapeutic agents not only for neurodegenerative diseases but also for non-alcoholic fatty liver disease and periodontal disease [119,120]. However, it is important to note that Nrf2 plays a multifaceted role in the occurrence and progression of cancer [121,122].

## Figures and Tables

**Table 1:** Nrf2 expression and its functional role in neurodegenerative diseases.

Disease	Nrf2 Expression in Patients	Nrf2 Knockout in Disease Models	Nrf2 Overexpression in Disease Models
AD	Decreased nuclear expression in hippocampal neurons [16].	Exacerbated cognitive decline and enhanced neurodegeneration in transgenic mouse models [17,18].	Lentivirus vector-mediated Nrf2 injection into the hippocampus improved spatial learning [19].
PD	Stronger expression in the nuclei of remaining dopaminergic neurons [16].	Enhanced dopaminergic cell death in response to 1-methyl-4-phenyl-1,2,3,6-tetrahydropyridine toxicity [20,21].	Astrocyte-specific Nrf2 overexpression rescued the loss of dopaminergic neurons in the substantia nigra and striatum [20].
MS	Upregulated in both the nucleus and cytoplasm of infiltrating macrophages in lesioned brain area [22].	More rapid onset and more severe clinical progression in the disease model [23,24].	Adeno-associated virus vector-mediated Nrf2 gene transfer did not suppress optic nerve inflammation or demyelination but protected retinal ganglion cells [25].
ALS	Decreased protein and mRNA expression in the motor cortex and spinal cord [26].	Mildly accelerated the age of onset and shortened lifespan in transgenic mouse models [27].	Overexpression of Nrf2 in astrocytes delayed disease onset and extended the lifespan of mice [28].
HD	[No data provided]	Increased vulnerability to mitochondrial complex II-induced lesions in the striatum [29].	Astrocytes overexpressing Nrf2 protected striatal neurons from mitochondrial complex II inhibition [30].
Friedreich’s ataxia	Decreased in fibroblasts and peripheral blood mononuclear cells isolated from patients, inhibiting nuclear translocation [31,32].	[No data provided]	[No data provided]

## Data Availability

The data that support the findings of this review are available from the corresponding author, upon reasonable request.
